# Genome‐wide data mining to construct a competing endogenous RNA network and reveal the pivotal therapeutic targets of Parkinson's disease

**DOI:** 10.1111/jcmm.16190

**Published:** 2020-12-15

**Authors:** Jing Zhang, Ruiying Chen, Fan Shi, Pan Yang, Kun Sun, Xiaojing Yang, Yulan Jin

**Affiliations:** ^1^ School of Public Health North China University of Science and Technology Tangshan China; ^2^ Department of neurology North China University of Science and Technology affiliated hospital Tangshan China

**Keywords:** biomarker, competing endogenous RNA, glutathione, Parkinson's disease, sphingolipid

## Abstract

Parkinson's disease (PD) is one of the most common neurodegenerative movement disorders, for which there has been no effective treatments. To clarify the pathogenesis of PD, we constructed a competing endogenous RNA (ceRNA) network based on the genome‐wide RNA sequencing data. It was found that 92 RNAs were differentially expressed, including 50 mRNAs, 25 miRNAs and 17 lncRNAs, based on which a ceRNA network was constructed and evaluated from 4 aspects of number of nodes, topological coefficients, closeness centrality and betweenness centrality. The functional annotation and enrichment analysis suggested that 6 functional modules, particularly the peripheral nervous system development and toxin metabolic process, dominated the development of PD. To validate the assumption, the gene set enrichment analysis (GSEA) was conducted basing on the genome‐wide RNAs regardless whether they were differentially expressed or not. Consistently, the results revealed that dysregulation of MAG, HOXB3, MYRF and PLP1 led to metabolic disorders of sphingolipid and glutathione, which contributed to the pathogenesis of PD. Also, in‐depth mining of previous literature confirmed a pivotal role of these dysregulated RNAs, which had been indicated to be potential diagnostic and therapeutic biomarkers of PD. Overall, we constructed a ceRNA network based on the dysregulated mRNAs, lncRNAs and miRNAs in PD, and the aberrant expression of MAG, HOXB3, MYRF and PLP1 caused metabolism disorder of sphingolipid and glutathione, and these genes are of great significance for the diagnosis and treatment of PD.

## 1 INTRODUCTION

Parkinson's disease (PD) is one of the most common neurodegenerative diseases, which is characterized by static tremor, bradykinesia, myotonia, postural balance disorder and cognitive impairment, and the cognitive impairment and gait dysfunction aggravate with the development of PD.^1^ Although clinical features of PD are apparent, its aetiology remains obscure. Given the potential cause of dopaminergic neuron death, a set of risk factors derived from genetics, environment, ageing and oxidative stress are indicated to contribute to PD.^2^ Currently, the treatment of PD mainly focuses on dopamine (DA) replacement and tremor relief, but there are many side effects such as the on‐off phenomenon.^3^ Moreover, these therapeutic strategies cannot prevent the development and evolution of neurodegeneration, which seriously affects the life quality of PD patients. On the other hand, available epidemiological studies reveal that 2.0% of people over 65 years old are affected by PD, and its morbidity is 1.5% higher in men than that of women.^4^ Worldwide, the incidence rate of the newly diagnosed PD is estimated to be 8 to 18 per 100,000 people yearly.^5^ The conditions are even less optimistic in primarily industrialized countries, where most of the PD patients are sporadic with less than 10% of family history, and the estimated PD prevalence ranges from 0.3% to 3.0% with the increase of age. For the highly industrialized regions such as Europe, the estimated PD prevalence is 65 to 12, 500 per 100,000 people, and the newly diagnosed PD cases are 346 per 100, 000 people every year.^6^ Considering the expanding population of PD, as well as the defects of the current therapeutic strategies, it is urgent to identify the potential therapeutic targets that will help to clarify PD pathogenesis.

Over the past 30 years, many standards and guidelines have been applied to optimize the diagnosis of PD. However, at current stage, it still needs opinions from the movement disorder specialists to determine the diagnosis.^7^, ^8^ Biomarkers are biochemical indicators reflecting changes of various physical disorders, and they can be objectively measured, evaluated and applied to the diagnosis of many diseases.^9^ Also, biomarkers are used to predict the occurrence, development and prognosis of diseases, and they play pivotal roles in indicating pharmacological response to specific treatment and intervention.^10^, ^11^ Given the current strategy for PD diagnosis, which mainly depends on clinical manifestations and often confounded by the atypical symptoms, identification of specific diagnostic biomarkers is of great significance for the diagnosis and treatment of PD.

Findings obtained in previous studies provide great help for screening potential diagnostic biomarkers of PD. It is found that the development of PD is a complicated and sophistically regulated biological process, in which mRNAs, miRNAs and lncRNAs are commonly investigated. miRNAs are dozens of highly conservative non‐coding small RNAs, and they can inhibit protein synthesis by interacting with protein‐coding or limited protein‐coding RNAs at the post‐transcriptional level. Several miRNAs have been indicated as potential biomarkers of PD.^12^ For example, upregulation of miR‐133b leads to silence of lncRNA SNHG14, whereas inadequate SNHG14 suppresses the expression of α‐syn that initiates the pathogenesis of PD.^13^ Considering the regulatory mechanism between mRNAs, miRNAs and lncRNAs, a competing endogenous RNA (ceRNA) network can be constructed, which facilities a comprehensive understanding of the pathogenesis of PD. In 2011, Salmone *et al* first proposed the hypothesis of the ceRNA network, and the hypothesis is based on the competition of a finite pool of miRNAs between different RNAs.^14^, ^15^ Given the base‐paring model between different RNAs, the ceRNA network is predicted by sequence alignment. Up to date, the ceRNA network has been applied to the diagnosis and treatment of numerous human diseases, especially for cancer and atherosclerosis.^16^, ^17^, ^18^ However, it has not been reported in PD.

Overall, we constructed a ceRNA network based on the genome‐wide RNA sequencing data of lncRNAs, miRNAs and mRNAs. The samples used to profile the RNA expression were obtained from the Gene Expression Omnibus (GEO), and a ceRNA network was built and evaluated using a statistical model. Besides, the biological function dominated by the ceRNA network was predicted from two aspects of Gene Ontology (GO) and Kyoto Encyclopedia of Genes and Genomes (KEGG). Also, the gene set enrichment analysis (GSEA) was implemented to reveal the molecular mechanism related to the functional changes of PD.

## 2 MATERIALS AND METHODS

### 2.1 Data source

All foundational data were downloaded from GEO, where the microarray and sequence‐based RNA profiles are free to access and available with no restrictions for research (https://www.ncbi.nlm.nih.gov/geo/). RNAs with abundance less than four degrees were excluded. The homogeneity of the samples was investigated by cluster analysis and principal component analysis, and the heterogeneous samples were removed. For mRNA and miRNA, 16 cingulate gyri from eight control and eight PD patients were collected and subjected to an Illumina HiSeq1000 instrument (1 x 50 bp) for high‐throughput sequencing, and the profiles were recruited in this study to construct the ceRNA network. LncRNAs were extracted from a Human Genome U133 PLUS 2.0 array according to our previous work,^19^ containing four samples of H4 human neuroglioma cells; two of the 4 samples were used to simulate PD by inducing mutant to upregulate aSyn tagged with *myc*; and the other two samples were wild‐type cells. Approval was obtained from the Institutional Review Board of North China University of Science and Technology (study number E1‐20191001).

### 2.2 Identification of the differentially expressed RNAs (DERNAs)

The differentially expressed mRNAs, miRNAs and lncRNAs were determined by Limma test, and a *P*‐value less than 0.05 and | log_2_ (fold change) | ≥ 1 were applied to screen DERNAs. Three heatmaps regarding the expression of these DERNAs were plotted using the pheatmap package in R software.

### 2.3 Construction of the mRNA‐DEmiRNA‐lncRNA triple network

According to the hypothesis of the ceRNA network, we examined all potential connections among the aberrantly expressed mRNAs, miRNAs and lncRNAs. Using miRNA as a bridge, we linked the DEmRNAs and DElncRNAs by searching the miRNA complementary sequences, and a triple network among DEmiRNAs and their targeted lncRNAs and mRNAs was constructed. The target genes of the DEmiRNAs were predicted using the microT scoring method (paring score ≥ 0.8) via the DIANA platform, and lncRNAs regulated by the DEmiRNAs were identified through alignment with lncBase Version 2.0. The Pearson correlation coefficient between lncRNAs and mRNAs was calculated, and only the pairs with coefficients more than 0.4 were involved in the triple network. Finally, a topological network of mRNAs‐DEmiRNAs‐lncRNAs was visualized using Cytoscape Version 3.7.1.

### 2.4 Construction of the ceRNA network

To construct the ceRNA network, we extracted the DEmRNAs and DElncRNAs targeted by DEmiRNAs from the triple network. By adopting the hypergeometric test, we evaluated the significance of the shared miRNAs between each DEmRNA and DElncRNA. A *P*‐value was calculated as follows and considered as statistically significant if it was less than 0.05:

$$P=1‐\sum_{t=0}^{x}\frac{\begin{array}{cc} \left(\begin{array}{c} t\\ K \end{array}\right) & \left(\begin{array}{c} \text{N - t}\\ \text{M - K} \end{array}\right) \end{array}}{\left(\begin{array}{c} N\\ M \end{array}\right)}$$

where K is the number of miRNAs interacted with mRNAs, and N is the number of miRNAs interacted with lncRNAs. M is the number of miRNAs in the genome, and *x* is the number of miRNAs shared by mRNAs and lncRNAs.^20^

### 2.5 Fitness assessment of the ceRNA network

The topological analysis of the ceRNA network was carried out by using the NetworkAnalyzer plugin in Cytoscape version 3.7.1.^21^ The validity of the model was studied from 4 aspects of number of nodes, topological coefficients, closeness centrality and betweenness centrality. The number of nodes was the number of edges that linked to this node. The topological coefficient was a relative measure for the extent to which a node shared neighbours with other nodes. Closeness centrality showed the connection of a node with other nodes using the standardized inverse average distance. Betweenness centrality was a measure of node in a network, which was the number of the shortest paths from each node to all others that passed through the node.^22^, ^23^ A *P*‐value less than 0.05 was considered as statistically significant.

### 2.6 Functional annotation and enrichment analysis

Based on RNAs in the triple network and ceRNA network, the functional annotation was performed from 4 aspects of biological process, molecular function, cellular component and signalling pathway. The analytic parameters for functional annotation were set as default in ClueGO and CluePedia in Cytoscape version 3.7.1. The EnrichmentMap package in Cytoscape was recruited to summarize the functional annotation items into different modules, and the hub targets were identified in the PD‐related functional modules. Specially, the hub targets were verified by the targeted GSEA regardless of whether the RNAs were differentially expressed or not.

### 2.7 Statistical analysis

All statistical analyses were performed using R software version 3.6.1 for Windows. The difference of RNA expression was compared between PD and the healthy control using Limma test. A Benjamin‐Hochberg adjusted *P*‐value < 0.05 was as considered statistically significant unless otherwise indicated.

## 3 RESULTS

### 3.1 Identification of the differentially expressed RNAs

**FIGURE 1:**
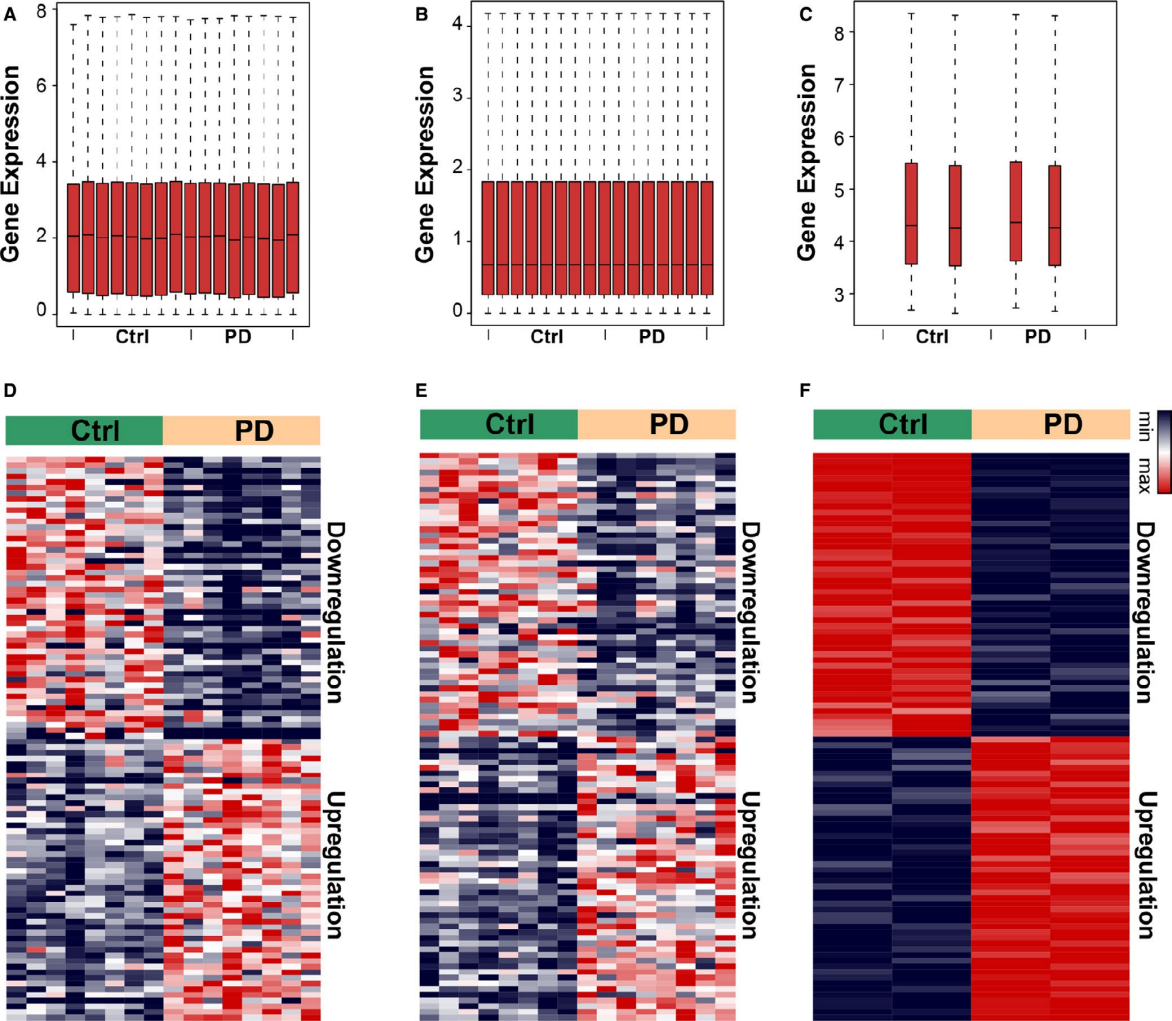
Identification of the differentially expressed RNAs. (A‐C) Box plots showing the expression of mRNAs, miRNAs and lncRNAs. (D‐F) Heatmap showing the differentially expressed mRNAs, miRNAs and lncRNAs. Red, upregulation; blue, downregulation. Ctrl, the healthy subjects; PD, Parkinson's disease

To investigate the relationship between different subtypes of RNAs in PD, we identified the dysregulated mRNAs, lncRNAs and miRNAs, respectively. In total, three data sets of mRNAs, lncRNAs and miRNAs of PD were retrieved from GEO: GSE110716, GSE95427 and GSE110719, where the expression of 22 278 mRNAs, 20 402 lncRNAs and 1014 miRNAs was detected, and 475 DEmRNAs, 142 DEmiRNA sand 40 DElncRNAs were determined after rigorous quality control (Figure 1). In detail, we found that 184 mRNAs, 17 lncRNAs and 60 miRNAs were upregulated, and 291 mRNAs, 23 lncRNAs and 82 miRNAs were downregulated. The top 10 dysregulated RNAs of different subtypes are listed in Table 1. These data suggested that suffering from PD altered the expression of mRNAs, lncRNAs and miRNAs.

**TABLE 1:** Top 10 dysregulated mRNAs, lncRNAs and miRNAs in Parkinson's disease

Gene_Symbol	log_2_(Fold Change)	Regulation	t Value	B Value	P‐Value
mRNAs					
GALNT5	−2.73	Down	−10.63	−1.60	3.41E‐05
LOC100129940	−2.60	Down	−7.37	−3.24	1.54E‐03
ONECUT2	−2.54	Down	−3.37	−1.83	3.85E‐03
AK094194	2.35	UP	5.60	−3.36	4.40E‐03
FNDC7	2.18	UP	3.27	−1.99	4.75E‐03
HMGB3P11	2.14	UP	5.12	−3.41	6.18E‐03
KRT18P33	−2.85	Down	−6.00	−3.67	7.92E‐03
DPRXP4	−2.39	Down	−3.49	−3.24	9.78E‐03
AC002558.1	2.94	UP	5.33	−3.71	1.12E‐02
AURKB	2.53	UP	3.88	−3.60	1.66E‐02
AK055397	−2.87	Down	−5.69	−4.07	2.46E‐02
LOC100128333	−2.55	Down	−4.01	−3.83	2.51E‐02
LOC100289495	2.82	UP	5.59	−4.07	2.55E‐02
AK128141	2.75	UP	5.46	−4.08	2.67E‐02
PERB11.3	2.82	UP	3.84	−3.85	2.83E‐02
AK022424	2.65	UP	5.25	−4.08	2.89E‐02
MCCD1	−2.65	Down	−5.24	−4.08	2.90E‐02
AC092953.1	−2.45	Down	−4.87	−4.09	3.38E‐02
FAM90A12	−3.82	Down	−2.38	−3.49	3.60E‐02
AK097064	2.37	UP	4.69	−4.10	3.64E‐02
miRNAs					
hsa‐miR‐4772‐5p	−2.19	Down	−8.75	7.16	1.39E‐07
hsa‐miR‐7157‐5p	2.79	Up	6.04	3.17	1.53E‐05
hsa‐miR‐5009‐5p	−2.21	Down	−5.26	1.80	7.15E‐05
hsa‐miR‐521	−1.99	Down	−4.34	0.08	4.78E‐04
hsa‐miR‐4518	2.46	Up	4.14	−0.73	1.28E‐03
hsa‐miR‐4653‐5p	2.33	Up	3.75	−1.46	3.02E‐03
hsa‐miR‐302b‐3p	2.63	Up	3.47	−1.66	3.33E‐03
hsa‐miR‐4499	−2.27	Down	−3.43	−1.82	4.27E‐03
hsa‐miR‐451b	−2.42	Down	−3.30	−1.94	4.37E‐03
hsa‐miR‐6728‐5p	2.42	Up	3.13	−2.27	6.33E‐03
hsa‐miR‐4268	2.33	Up	3.05	−2.43	7.50E‐03
hsa‐miR‐520a‐5p	2.03	Up	3.02	−2.49	8.01E‐03
hsa‐miR‐494‐5p	−2.09	Down	−2.81	−2.87	1.23E‐02
hsa‐miR‐298	−2.14	Down	−2.78	−3.01	1.87E‐02
hsa‐miR‐5580‐5p	2.00	Up	2.82	−3.11	2.45E‐02
hsa‐miR‐5589‐5p	−2.05	Down	−2.54	−3.39	2.88E‐02
hsa‐miR‐6718‐5p	−1.92	Down	−2.35	−3.71	3.17E‐02
hsa‐miR‐4700‐3p	2.33	Up	2.30	−3.78	3.57E‐02
hsa‐miR‐5008‐5p	2.26	Up	2.30	−3.75	3.95E‐02
hsa‐miR‐4752	−2.26	Down	−2.23	−3.90	4.13E‐02
lncRNAs					
PURPL	2.25	Up	22.54	6.71	8.52E‐07
GAS5	−2.02	Down	−22.52	6.71	8.56E‐07
LINC01638	2.09	Up	19.23	5.91	2.08E‐06
RMST	1.98	Up	16.39	5.05	5.11E‐06
LINC00662	−1.62	Down	−15.96	4.90	5.95E‐06
SNHG26	2.10	Up	15.57	4.77	6.81E‐06
LINC01133	−1.56	Down	−15.13	4.61	8.01E‐06
LINC00944	−1.57	Down	−13.97	4.15	1.25E‐05
MIR3142HG	−2.77	Down	−13.33	3.89	1.62E‐05
AC144831.1	1.17	Up	13.14	3.81	1.75E‐05
LINC01419	1.21	Up	12.63	3.57	2.19E‐05
PWAR6	1.25	Up	11.99	3.27	2.91E‐05
AC103740.2	1.45	Up	11.32	2.94	4.00E‐05
LINC01119	1.41	Up	11.21	2.88	4.21E‐05
LINC00326	−1.77	Down	−10.69	2.60	5.47E‐05
DIRC3	1.52	Up	9.94	2.17	8.14E‐05
LINC00662	−1.47	Down	−9.47	1.88	1.06E‐04
SNHG3	−1.34	Down	−8.99	1.58	1.40E‐04
MIR100HG	−1.29	Down	−8.63	1.33	1.74E‐04

### 3.2 Construction of lncRNA‐DEmiRNA‐mRNA Triple Network in PD

To investigate the interaction between different subtypes of RNAs in PD, we constructed a triple network using DEmiRNAs as a bridge for mRNAs and lncRNAs, and identified the putative interactive pairs of DEmiRNA‐mRNA and DEmiRNA‐lncRNA using R software. In total, 6006 mRNAs and 17 850 lncRNAs targeted by 142 DEmiRNAs were identified (Supplementary Figure S1 and S2), and then, 137 miRNAs targeted by both mRNAs and lncRNAs were extracted to construct the lncRNA‐DEmiRNA‐mRNA triple network. Also, 4235 mRNAs and 7728 lncRNAs were involved in the final model (Supplementary Figure S3). The functional enrichment analysis was conducted to explore the practical implication of the triple network from 4 aspects of biological process, molecular function, cellular component and signalling pathway. Interestingly, we found that the triple network was supposed to play crucial roles in the pathogenesis of neurodegenerative diseases and could be used to guide further studies.

### 3.3 Construction and fitness assessment of ceRNA Network in PD

**FIGURE 2:**
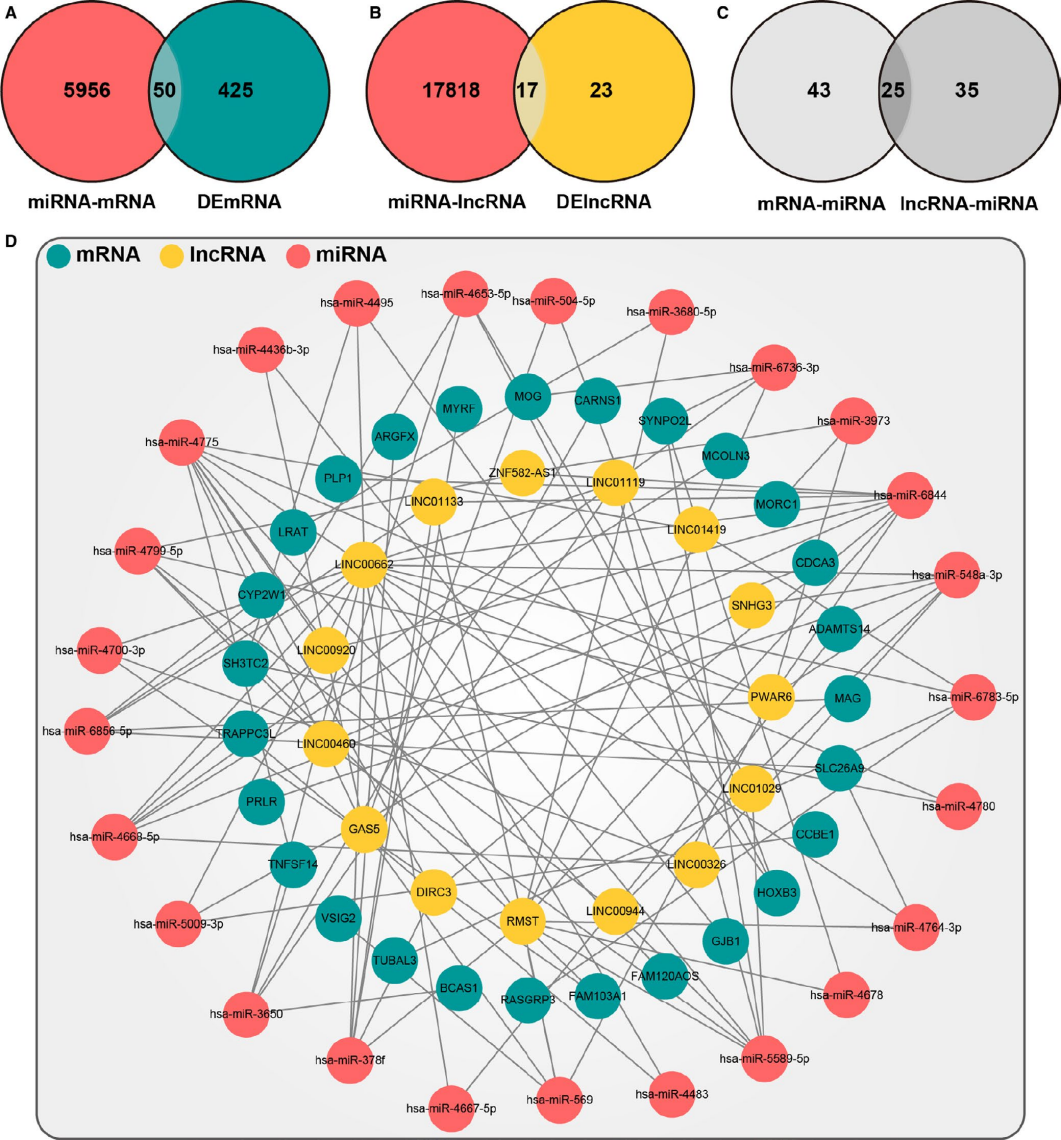
The layout of the ceRNA network. (A) Venn diagram of the differentially expressed mRNAs (DEmRNAs) in the ceRNA network. These mRNAs distributed in the overlap region of mRNAs targeted by DEmiRNAs and DEmRNAs in Parkinson's disease (PD). (B) Venn diagram of DElncRNAs in the ceRNA network. These lncRNAs distributed in the overlap region of lncRNAs targeted by DEmiRNAs and DElncRNAs in PD. (C) Venn diagram of miRNAs in the ceRNA network in PD. These miRNAs distributed in the overlap region of DEmiRNAs targeted by the overlap mRNAs in (A) or lncRNA in (B). (D) ceRNA network constructed by miRNAs, lncRNAs and mRNAs obtained from the overlap regions of (A), (B) and (C)

**FIGURE 3:**
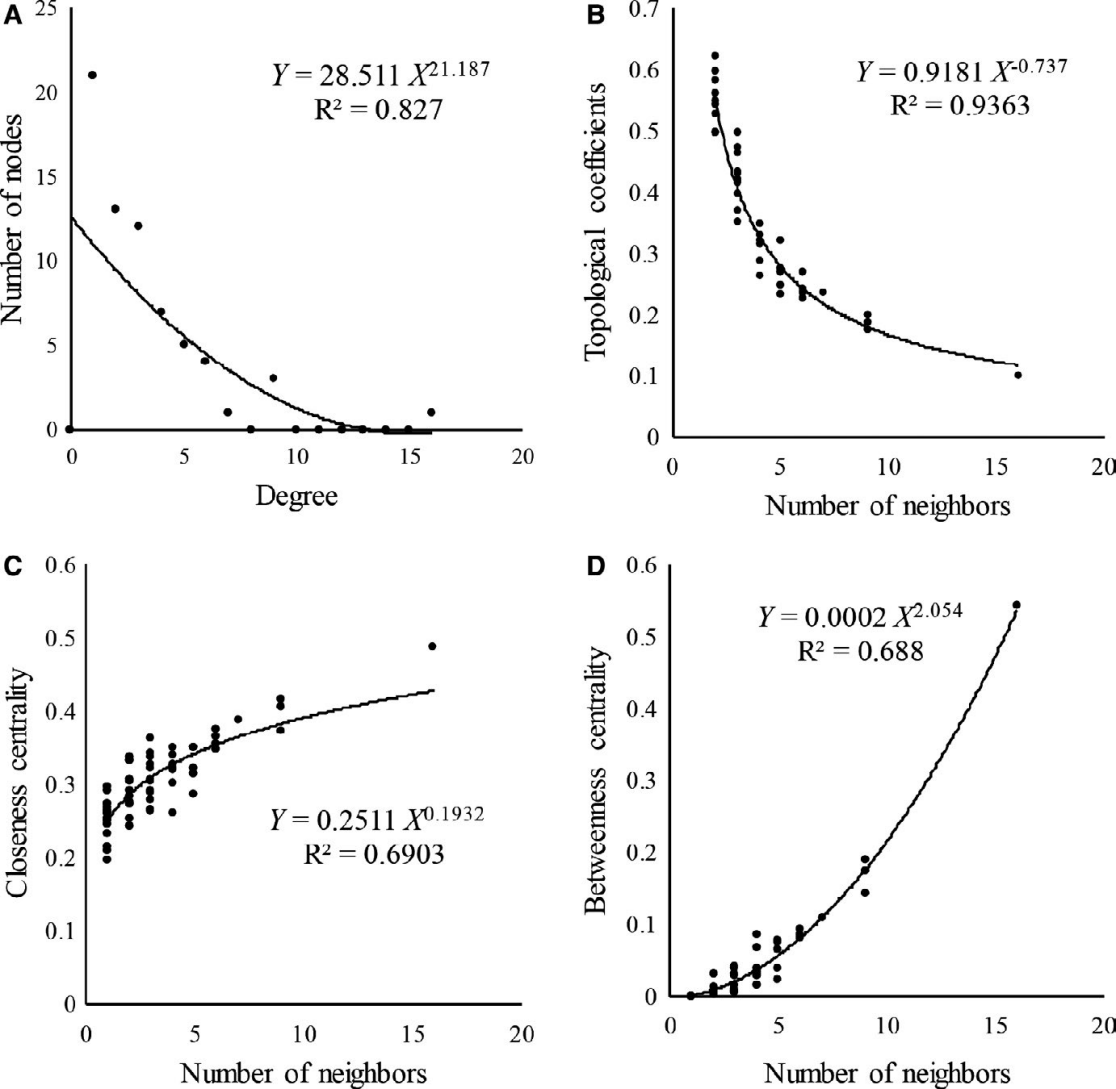
Fitness assessment of the ceRNA network. (A‐D) Assessment of the ceRNA network from 4 aspects of the number of nodes (A), topological coefficients (B), closeness centrality (C) and betweenness centrality (D). The values of R‐squares of the number of nodes, topological coefficient, closeness centrality and betweenness centrality were 0.827,0.936,0.690 and 0.688, indicating a highly efficient ceRNA network was constructed

To construct the ceRNA network in PD, we extracted DEmiRNAs, DEmRNAs and DElncRNAs in the triple network. Using the hypergeometric test, we built the ceRNA network consisted of 27 DEmRNAs, 15 DElncRNAs and 25 DEmiRNAs, where 99 DEmRNA‐DElncRNA‐DEmiRNA interactive pairs were identified and are shown in Figure 2 and Table 2. Moreover, we performed the topological assessment to evaluate the fitness of the ceRNA network at node degree, topological coefficient, closeness centrality and betweenness centrality. As shown in Figure 3, the square coefficients of node degree, topological coefficient, closeness centrality and betweenness centrality were 0.827, 0.936, 0.690 and 0.688, indicating that a highly convincing ceRNA network was successfully constructed.

**TABLE 2:** RNAs in the putative ceRNA network in Parkinson's disease

ID	mRNA	miRNA	lncRNA	ID	mRNA	miRNA	lncRNA
1	ADAMTS14	hsa‐miR‐4667‐5p	LINC00662	51	MYRF	hsa‐miR‐378f	RMST
2	ARGFX	hsa‐miR‐378f	DIRC3	52	PLP1	hsa‐miR‐6844	DIRC3
3	ARGFX	hsa‐miR‐378f	LINC00662	53	PLP1	hsa‐miR‐6844	LINC00460
4	ARGFX	hsa‐miR‐378f	LINC01133	54	PLP1	hsa‐miR‐6844	LINC00662
5	ARGFX	hsa‐miR‐378f	RMST	55	PLP1	hsa‐miR‐6844	LINC00920
6	BCAS1	hsa‐miR‐3650	LINC00662	56	PLP1	hsa‐miR‐6844	LINC01133
7	BCAS1	hsa‐miR‐3650	LINC01119	57	PLP1	hsa‐miR‐6844	PWAR6
8	CARNS1	hsa‐miR‐3650	LINC00662	58	PLP1	hsa‐miR‐6844	RMST
9	CARNS1	hsa‐miR‐3650	LINC01119	59	PLP1	hsa‐miR‐6844	ZNF582‐AS1
10	CCBE1	hsa‐miR‐5009‐3p	LINC00662	60	PRLR	hsa‐miR‐548a‐3p	LINC00326
11	CCBE1	hsa‐miR‐5009‐3p	SNHG3	61	PRLR	hsa‐miR‐548a‐3p	LINC00662
12	CDCA3	hsa‐miR‐4668‐5p	LINC00326	62	PRLR	hsa‐miR‐548a‐3p	LINC00944
13	CDCA3	hsa‐miR‐4668‐5p	LINC00662	63	PRLR	hsa‐miR‐548a‐3p	PWAR6
14	CDCA3	hsa‐miR‐4668‐5p	LINC00920	64	PRLR	hsa‐miR‐548a‐3p	SNHG3
15	CDCA3	hsa‐miR‐4668‐5p	LINC01119	65	RASGRP3	hsa‐miR‐6783‐5p	LINC00662
16	CYP2W1	hsa‐miR‐6856‐5p	LINC00662	66	RASGRP3	hsa‐miR‐6783‐5p	LINC01419
17	CYP2W1	hsa‐miR‐6856‐5p	LINC01133	67	SH3TC2	hsa‐miR‐4495	LINC00662
18	FAM103A1	hsa‐miR‐4700‐3p	LINC00326	68	SH3TC2	hsa‐miR‐4653‐5p	LINC00460
19	FAM103A1	hsa‐miR‐4700‐3p	LINC00662	69	SH3TC2	hsa‐miR‐4653‐5p	LINC01029
20	FAM103A1	hsa‐miR‐4700‐3p	LINC01119	70	SH3TC2	hsa‐miR‐4780	LINC00662
21	FAM103A1	hsa‐miR‐4799‐5p	LINC00460	71	SLC26A9	hsa‐miR‐4764‐3p	LINC00662
22	FAM103A1	hsa‐miR‐4799‐5p	LINC00944	72	SLC26A9	hsa‐miR‐4764‐3p	RMST
23	FAM103A1	hsa‐miR‐4799‐5p	PWAR6	73	SLC26A9	hsa‐miR‐6856‐5p	LINC00662
24	FAM103A1	hsa‐miR‐4799‐5p	ZNF582‐AS1	74	SLC26A9	hsa‐miR‐6856‐5p	LINC01133
25	FAM120AOS	hsa‐miR‐4775	DIRC3	75	SYNPO2L	hsa‐miR‐4678	RMST
26	FAM120AOS	hsa‐miR‐4775	GAS5	76	SYNPO2L	hsa‐miR‐5589‐5p	LINC00460
27	FAM120AOS	hsa‐miR‐4775	LINC00662	77	SYNPO2L	hsa‐miR‐5589‐5p	LINC00662
28	FAM120AOS	hsa‐miR‐4775	LINC00920	78	SYNPO2L	hsa‐miR‐5589‐5p	LINC00944
29	FAM120AOS	hsa‐miR‐4775	LINC01419	79	SYNPO2L	hsa‐miR‐5589‐5p	LINC01029
30	FAM120AOS	hsa‐miR‐4775	PWAR6	80	SYNPO2L	hsa‐miR‐5589‐5p	LINC01119
31	FAM120AOS	hsa‐miR‐4775	RMST	81	SYNPO2L	hsa‐miR‐5589‐5p	RMST
32	GJB1	hsa‐miR‐4436b‐3p	GAS5	82	SYNPO2L	hsa‐miR‐6736‐3p	LINC01119
33	HOXB3	hsa‐miR‐4495	LINC00662	83	SYNPO2L	hsa‐miR‐6736‐3p	RMST
34	HOXB3	hsa‐miR‐4653‐5p	LINC00460	84	TNFSF14	hsa‐miR‐3650	LINC00662
35	HOXB3	hsa‐miR‐4653‐5p	LINC01029	85	TNFSF14	hsa‐miR‐3650	LINC01119
36	HOXB3	hsa‐miR‐504‐5p	GAS5	86	TNFSF14	hsa‐miR‐4775	DIRC3
37	LRAT	hsa‐miR‐3680‐5p	RMST	87	TNFSF14	hsa‐miR‐4775	GAS5
38	MAG	hsa‐miR‐6856‐5p	LINC00662	88	TNFSF14	hsa‐miR‐4775	LINC00662
39	MAG	hsa‐miR‐6856‐5p	LINC01133	89	TNFSF14	hsa‐miR‐4775	LINC00920
40	MCOLN3	hsa‐miR‐4668‐5p	LINC00326	90	TNFSF14	hsa‐miR‐4775	LINC01419
41	MCOLN3	hsa‐miR‐4668‐5p	LINC00662	91	TNFSF14	hsa‐miR‐4775	PWAR6
42	MCOLN3	hsa‐miR‐4668‐5p	LINC00920	92	TNFSF14	hsa‐miR‐4775	RMST
43	MCOLN3	hsa‐miR‐4668‐5p	LINC01119	93	TRAPPC3L	hsa‐miR‐4483	LINC00662
44	MOG	hsa‐miR‐6736‐3p	LINC01119	94	TUBAL3	hsa‐miR‐6783‐5p	LINC00662
45	MOG	hsa‐miR‐6736‐3p	RMST	95	TUBAL3	hsa‐miR‐6783‐5p	LINC01419
46	MORC1	hsa‐miR‐3973	PWAR6	96	VSIG2	hsa‐miR‐569	GAS5
47	MORC1	hsa‐miR‐3973	ZNF582‐AS1	97	VSIG2	hsa‐miR‐569	LINC01133
48	MYRF	hsa‐miR‐378f	DIRC3	98	VSIG2	hsa‐miR‐569	PWAR6
49	MYRF	hsa‐miR‐378f	LINC00662	99	VSIG2	hsa‐miR‐569	RMST
50	MYRF	hsa‐miR‐378f	LINC01133				

### 3.4 Functional annotation and enrichment analysis of the ceRNA network and identification of the hub genes in PD

**FIGURE 4:**
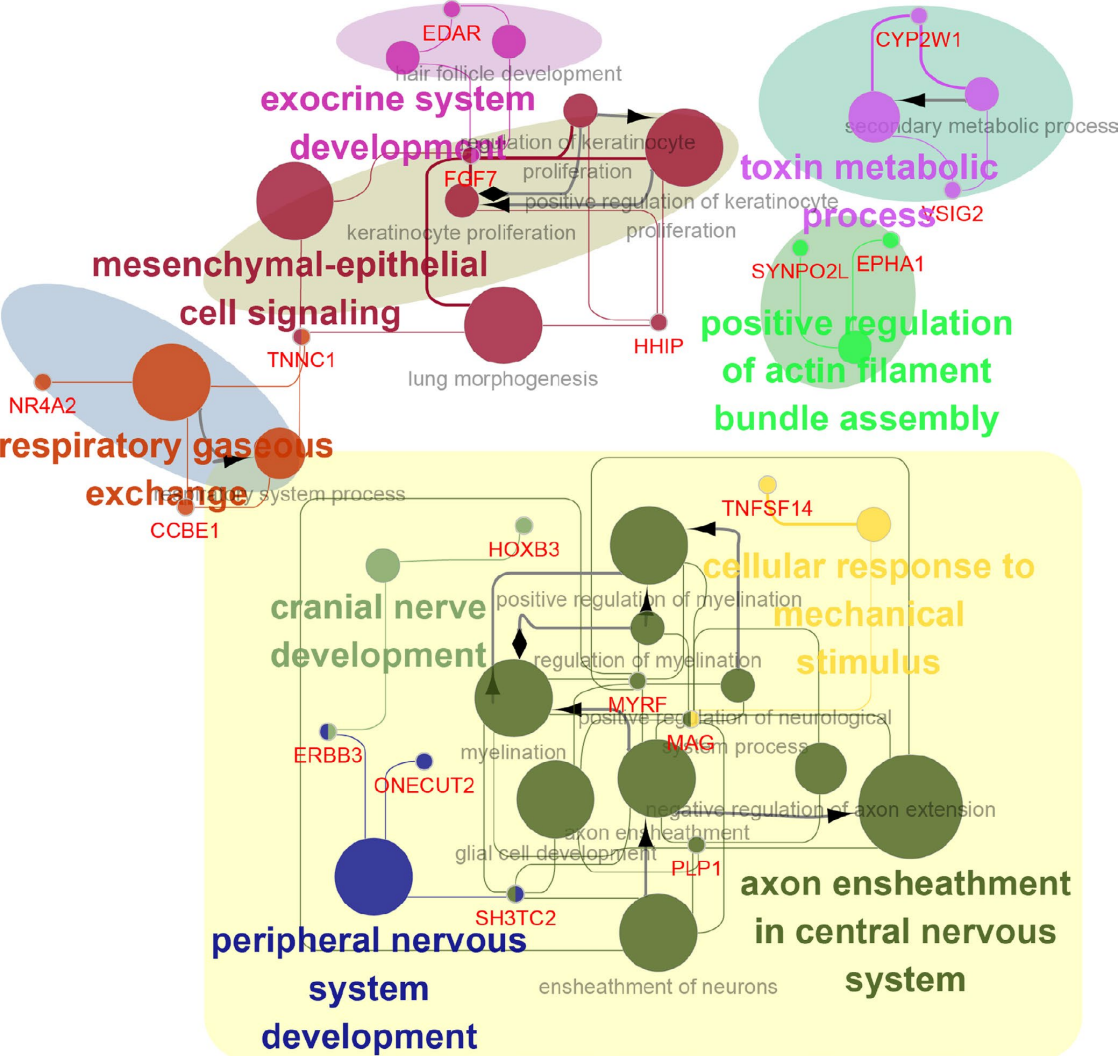
Functional annotation and enrichment analysis for RNAs involved in the ceRNA network. The total of nine functional modules was represented in different colours, and the nervous system‐related functional modules, along with their first neighbourhoods in the topological network, were painted with the yellow background

To explore the biological function dominated by the ceRNA network, we implemented functional annotation for the RNAs in the ceRNA network. As shown in Figure 4 and Supplementary Table S1, a total of 9 functional modules were identified, including mesenchymal‐epithelial cell signalling, cranial nerve development, peripheral nervous system development, axon ensheathment in the central nervous system, toxin metabolic process, exocrine system development, respiratory gaseous exchange and cellular response to mechanical stimulus. Thus, the nervous system‐related functional modules, along with their first neighbourhoods in the topological network, were recruited in the follow‐up study. In detail, 8 DEmRNAs, including HOXB3, ERBB3, ONECUT2, SH3TC2, PLP1, MAG, TNFSF14 and MYRF, were involved in 4 nervous system‐related functional modules of cranial nerve development, peripheral nervous system development, axon ensheathment in the central nervous system and cellular response to mechanical stimulus. In combination with evidence reported previously, these DEmRNAs as well as their related DEmiRNAs and DElncRNAs were most likely to mediate the pathogenesis of PD. Thus, it is speculated that dysregulation of HOXB3, ERBB3, ONECUT2, SH3TC2, PLP1, MAG, TNFSF14 and MYRF induces disorder in cranial nerve development, peripheral nervous system development, axon ensheathment in the central nervous system and cellular response to mechanical stimulus, which contributes to the pathogenesis of PD.

### 3.5 Validation of the crucial role of MAG/HOXB3/MYRF/PLP1‐related ceRNA network in the pathogenesis of PD

**FIGURE 5:**
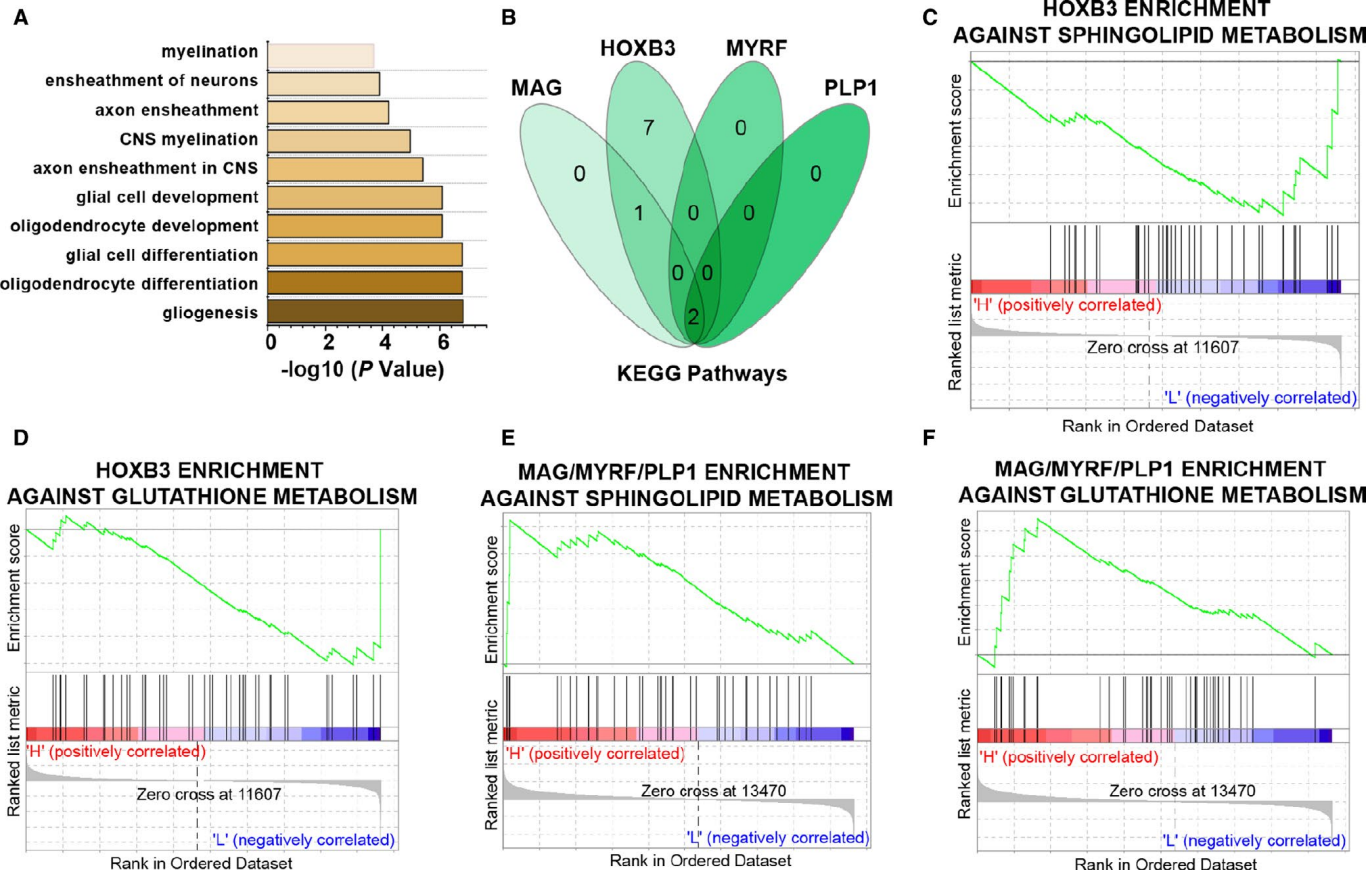
Gene set enrichment analysis (GSEA) identifies the hub genes and their related signalling pathways in Parkinson's disease. (A) Functional annotation and enrichment analysis of genes in the nervous system‐related functional modules. (B) Venn diagram showing the signalling pathways associated with MAG, HOXB3, MYRF and PLP1. (C‐F) MAG, HOXB3, MYRF and PLP1 enrichment against sphingolipid/glutathione metabolism by GSEA

To study the functional implication of the 8 hub genes in the ceRNA network, the functional annotation and enrichment analysis were performed. As shown in Figure 5 and Supplementary Table S2, these genes were predicted to regulate the biological processes associated with gliogenesis, oligodendrocyte differentiation, axon ensheathment and myelination. Moreover, the targeted GSEA was performed to examine the role of the signalling pathways related to these 8 hub genes in the development of PD. In total, MAG, HOXB3, MYRF and PLP1 were found to regulate sphingolipid and glutathione metabolism signalling pathway. However, based on the expression of these 4 DEmRNAs, the signalling pathway enrichment trend of HOXB3 was negatively correlated with that in MAG, MYRF and PLP1, indicating that MAG, HOXB3, MYRF and PLP1 might act oppositely in the pathogenesis of PD. These data suggest that aberrant expression of MAG, HOXB3, MYRF and PLP1 promotes PD via dysregulating sphingolipid and glutathione metabolism.

## 4 DISCUSSION

PD is one of the most common multifactorial neurodegenerative disorders, which mainly occurs in middle‐aged and elder people over 65 years old.^24^, ^25^ It is generally believed that PD is caused by genetic alteration, age and environment,^26^ yet the pathogenesis of PD has not been fully elucidated, which consequently calls into questions for its diagnosis and clinical treatments. With the rapid development of transcriptomics, the RNA interaction in PD has drawn considerable attentions. For example, it was found that TRPC6 in the blood was significantly suppressed in patients with mild cognitive impairment and could be used as a potential indicator for PD or Alzheimer's disease.^27^ Similarly, 8 miRNAs, including miR‐9‐5p, miR‐21‐5p, the miR‐29 family, miR‐132‐3p, miR‐124‐3p, miR‐146a‐5p, miR‐155‐5p and miR‐223‐3p, have been well‐studied in PD or PD models.^28^ More importantly, the dysregulation of miR‐133b by interfering lncRNA SNHG14 alters the α‐synuclein pathway and lead to mitigation of the dopaminergic neuron injury in PD.^13^ Therefore, a comprehensive investigation of the RNA interactions is conducive to the mechanistic study as well as the clinical treatment of PD.

Here, we recombined a data set using publicly available data sets of mRNAs, lncRNAs and miRNAs in PD, and then, we constructed a triple network that was further used to build the ceRNA network (DEmRNA‐DEmiRNA‐DElncRNA). The functional enrichment analysis revealed the potential regulatory mechanism of PD, where the signalling pathways of myelination and the formation and development of glial cells were previously confirmed to promote PD.^29^ Specially, we evaluated the fitness of the ceRNA network using R‐squares of the number of nodes, topological coefficient, closeness centrality and betweenness centrality. We found that the ceRNA network was of excellent statistical efficiency. Finally, a set of hub genes, including MAG, HOXB3, MYRF and PLP1, were determined using GSEA and predicted to mediate the sphingolipid and glutathione metabolism signalling pathways. Generally, sphingolipids are abundant in the central nervous system, and glutathione is an important antioxidant and free radical scavenger in vivo, both sphingolipid and glutathione are considered as crucial regulators of PD.

Since there are very few studies concerning the regulatory mechanism of ceRNA in PD, it is difficult to provide evidence for the speculated ceRNA network. However, several RNAs in the ceRNA network have been investigated, and the biological processes indicated by the functional annotation procedure are consistent with the pathogenesis of PD. For example, axon ensheathment in the central nervous system regulates the synthesis of the myelin sheath, which can elevate the speed of nerve excitation and ensure its directional transmission.^30^ Glial cells are widely distributed in the central nervous system, which supports and nourishes neurons, as well as absorbs the active substances, so the dysfunction of these biological activities is essential for the development of PD.^31^ For the hub genes in the ceRNA network, they were both validated by the GSEA and previous experiments. By inducing apoptosis, MAG was suggested to regulate the growth of cerebellar granule neurons (CGNs).^32^ As a member of the HOXB family, HOXB3 was in charge of cell differentiation and proliferation.^33^ Besides, MYRF and PLP1 were essential for the oligodendrocytes (OLS) differentiation and myelin maintenance in the central nervous system, which was tightly associated with long‐distance and rapid transmission of nerve electrical impulses.^34^, ^35^, ^36^ Consistently, we found that the signalling pathways such as myelination and the biosynthesis of differentiation and development of glial cells could promote the pathogenesis of PD. Here, in combination of previous studies, a total of 4 genes along with their corresponding DEmiRNAs and DElncRNAs were identified as potential diagnostic and therapeutic targets of PD.

Also, there were several limitations restricting the interpretation of the ceRNA network. Since the tissues used to identify the dysregulated lncRNAs were not in accordance with those used to determine DEmRNAs or DEmiRNAs, this could lead to the instability or uncertainty of the ceRNA network. The sample sizes of both tissues and PD cell models were limited, which might cause type II errors statistically. The ceRNA constructed in study was only validated using GSEA, so experiments should be strictly designed and conducted according to the ceRNA network in future studies.

In conclusion, we constructed a highly convincing ceRNA network based on the genome‐wide expression profiles of mRNAs, miRNAs and lncRNAs. The functional enrichment analysis and GSEA showed that dysregulation of MAG, HOXB3, MYRF and PLP1, as well as their corresponding miRNAs and lncRNAs in the ceRNA network, contributes to the pathogenesis of PD via sphingolipid and glutathione metabolism signalling pathway, and these RNAs of interest were potential diagnostic and therapeutic targets of PD.

## CONFLICTS OF INTEREST

The authors confirm that there are no conflicts of interest.

## AUTHOR CONTRIBUTIONS

**Jing Zhang:** Conceptualization (equal); Writing‐original draft (equal). **Ruiying Chen:** Data curation (equal); Formal analysis (equal); Investigation (equal). **Fan Shi:** Investigation (equal); Methodology (equal). **Pan Yang:** Validation (equal); Visualization (equal). **Kun Sun:** Resources (equal); Validation (equal); Visualization (equal). **Xiaojing Yang:** Resources (equal); Software (equal). **Yulan Jin:** Writing‐review & editing (equal).

## Supporting information

Fig S1

Fig S2

Fig S3

Table S1‐S2

## Data Availability

The data that support the findings of this study are openly available in the Gene Expression Omnibus at https://www.ncbi.nlm.nih.gov/geo/, reference number GSE110716, GSE 95 427 and GSE110719, and the supplementary material of this article.
