# Supplementary figures and images for: Genome‐wide data mining to construct a competing endogenous RNA network and reveal the pivotal therapeutic targets of Parkinson's disease
Source: J Cell Mol Med. 2020 Dec 15;25(13):5912–23. doi: 10.1111/jcmm.16190 (PMC8256352; doi:10.1111/jcmm.16190)

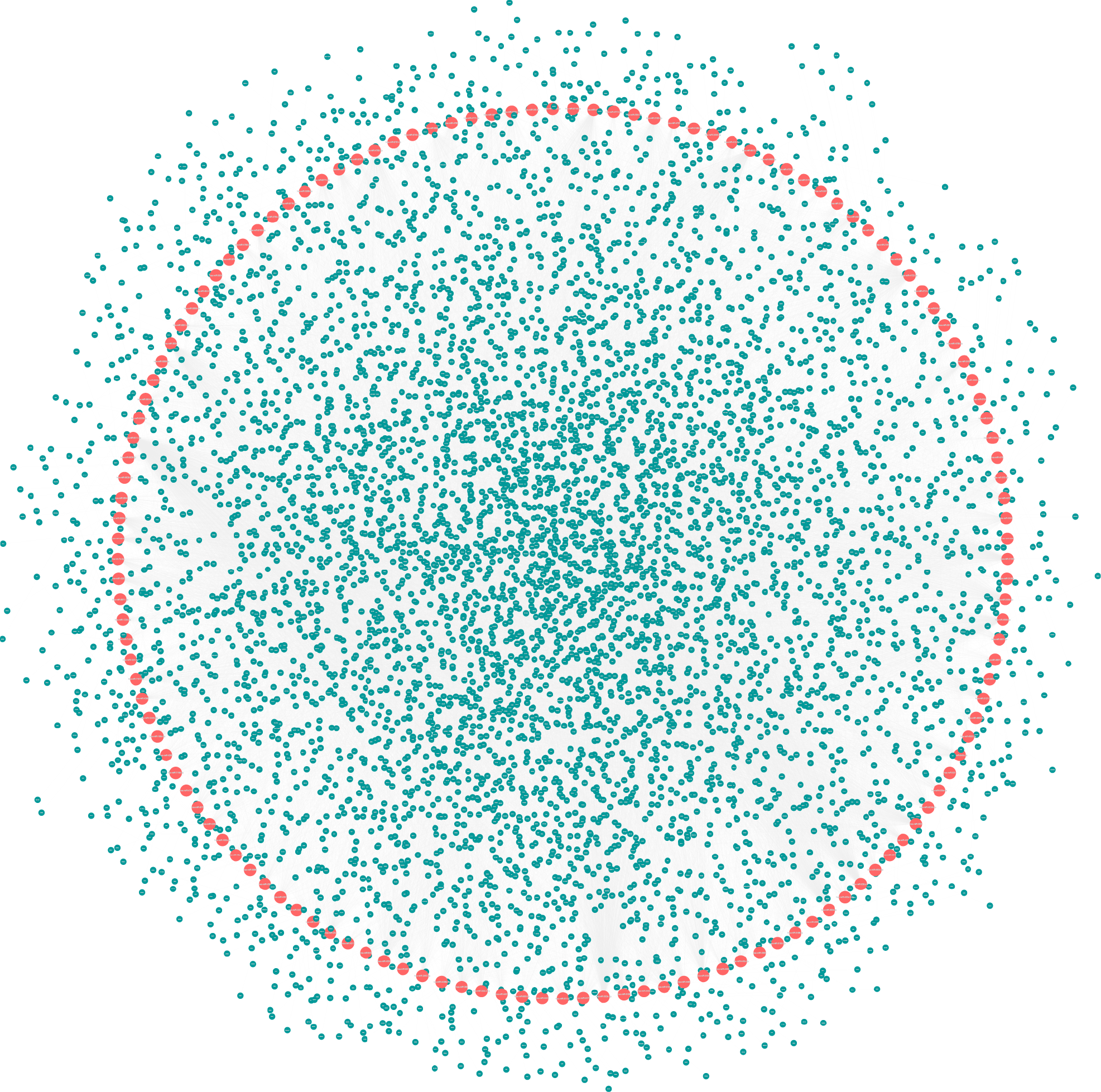

Supplement: Supplementary file 1 — Fig S1 [file JCMM-25-5912-s002.tif]

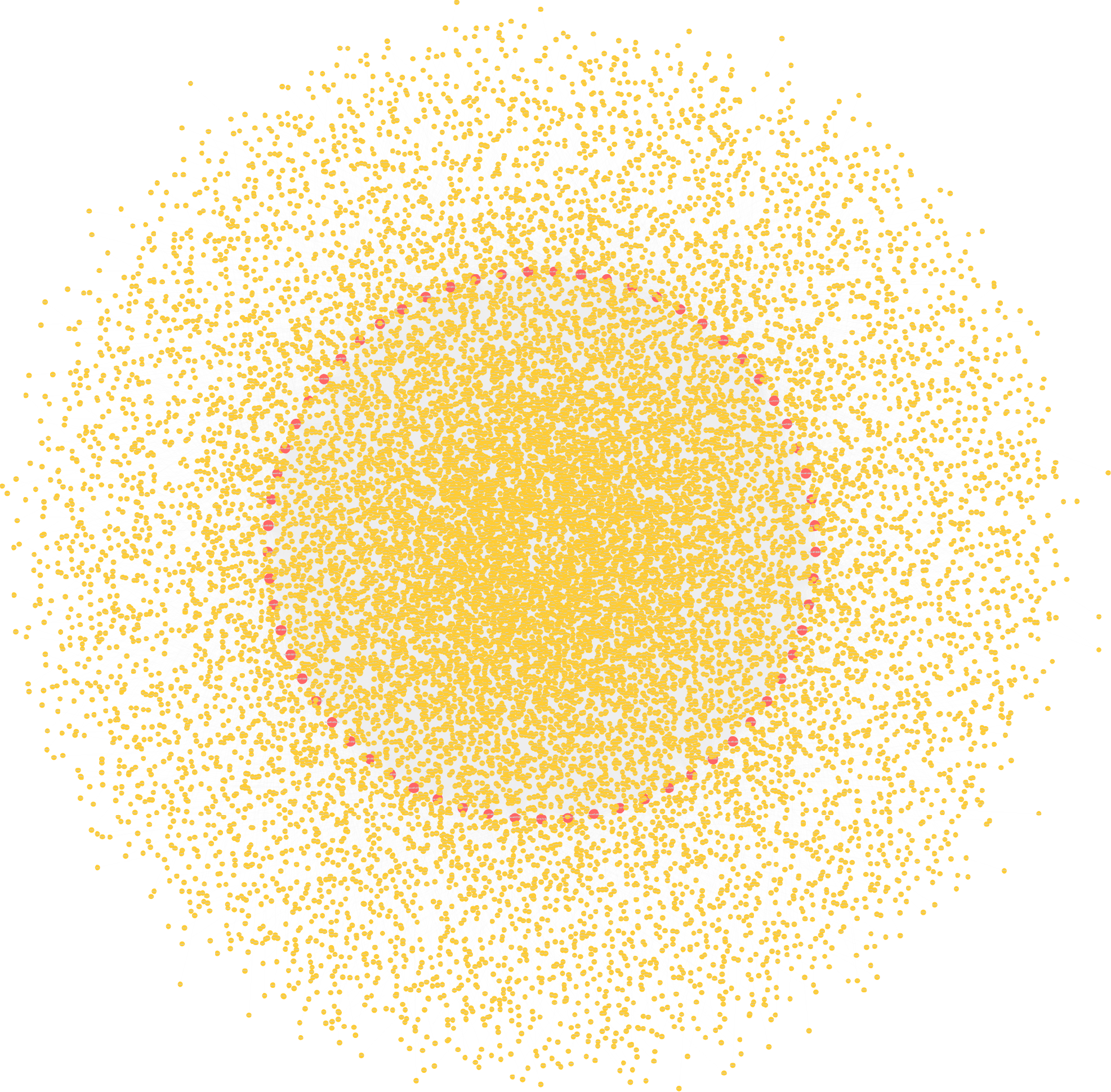

Supplement: Supplementary file 2 — Fig S2 [file JCMM-25-5912-s004.tif]

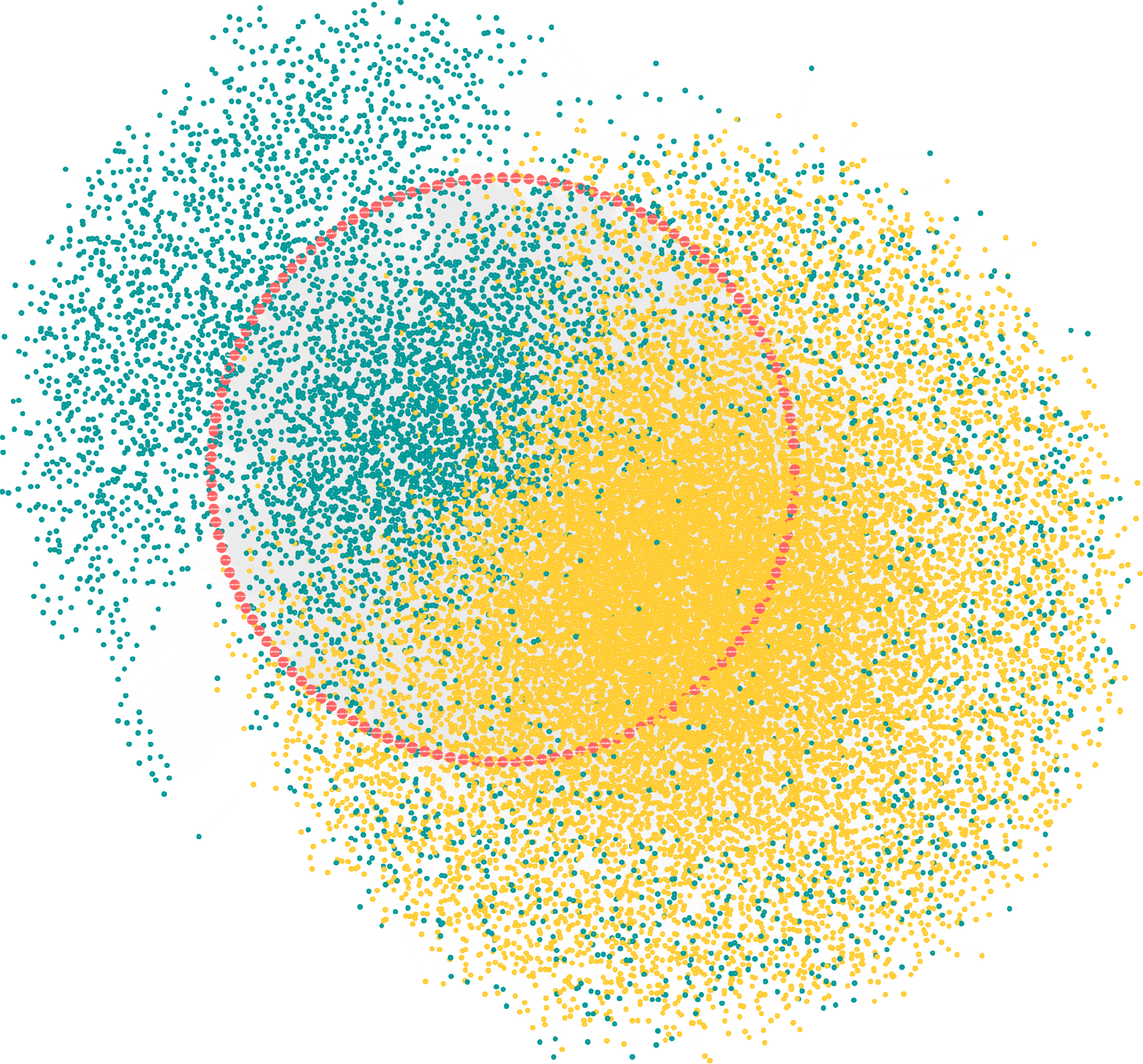

Supplement: Supplementary file 3 — Fig S3 [file JCMM-25-5912-s001.tif]
