# Supplementary material for: Genome‐wide data mining to construct a competing endogenous RNA network and reveal the pivotal therapeutic targets of Parkinson's disease
Source: J Cell Mol Med. 2020 Dec 15;25(13):5912–23. doi: 10.1111/jcmm.16190 (PMC8256352; doi:10.1111/jcmm.16190)
Supplement: Supplementary file 4 — Table S1‐S2 [file JCMM-25-5912-s003.docx]

**SUPPLEMENTARY TABLES**

**Table S1. Gene Ontology annotation of differentially expressed mRNAs in Parkinson’s Disease**

| **ID** | **GO ID** | **GO Term** | **P Value** | **Associated Genes (%)** | **Associated Genes Found** |
| --- | --- | --- | --- | --- | --- |
| 1 | GO:0021545 | Cranial nerve development | 1.37E-02 | 2.94 | ERBB3, HOXB3 |
| 2 | GO:0032233 | Positive regulation of actin filament bundle assembly | 1.95E-02 | 2.44 | EPHA1, SYNPO2L |
| 3 | GO:0071260 | Cellular response to mechanical stimulus | 2.67E-02 | 2.06 | MAG, TNFSF14 |
| 4 | GO:0007422 | Peripheral nervous system development | 1.91E-03 | 3.19 | ERBB3, ONECUT2, SH3TC2 |
| 5 | GO:0035272 | Exocrine system development | 1.19E-02 | 3.17 | EDAR, FGF7 |
| 6 | GO:0001942 | Hair follicle development | 2.83E-02 | 2.00 | EDAR, FGF7 |
| 7 | GO:0007585 | Respiratory gaseous exchange | 1.20E-03 | 3.75 | CCBE1, NR4A2, TNNC1 |
| 8 | GO:0003016 | Respiratory system process | 4.00E-03 | 5.56 | CCBE1, TNNC1 |
| 9 | GO:0019748 | Secondary metabolic process | 2.77E-02 | 2.02 | CYP2W1, VSIG2 |
| 10 | GO:0009404 | Toxin metabolic process | 3.17E-03 | 6.25 | CYP2W1, VSIG2 |
| 11 | GO:0043616 | Keratinocyte proliferation | 1.30E-02 | 3.03 | FGF7, HHIP |
| 12 | GO:0060638 | Mesenchymal-epithelial cell signaling | 4.36E-04 | 16.67 | FGF7, TNNC1 |
| 13 | GO:0010837 | Regulation of keratinocyte proliferation | 9.46E-03 | 3.57 | FGF7, HHIP |
| 14 | GO:0010838 | Positive regulation of keratinocyte proliferation | 1.50E-03 | 9.09 | FGF7, HHIP |
| 15 | GO:0060425 | Lung morphogenesis | 6.28E-04 | 4.69 | FGF7, HHIP, TNNC1 |
| 16 | GO:0007272 | Ensheathment of neurons | 8.45E-04 | 2.47 | MAG, MYRF, PLP1, SH3TC2 |
| 17 | GO:0031646 | Positive regulation of neurological system process | 1.57E-02 | 2.74 | MAG, MYRF |
| 18 | GO:0008366 | Axon ensheathment | 8.45E-04 | 2.47 | MAG, MYRF, PLP1, SH3TC2 |
| 19 | GO:0031641 | Regulation of myelination | 7.02E-03 | 4.17 | MAG, MYRF |
| 20 | GO:0032291 | Axon ensheathment in central nervous system | 5.87E-05 | 10.34 | MAG, MYRF, PLP1 |
| 21 | GO:0042552 | Myelination | 7.88E-04 | 2.52 | MAG, MYRF, PLP1, SH3TC2 |
| 22 | GO:0021782 | Glial cell development | 3.80E-04 | 3.05 | MAG, MYRF, PLP1, SH3TC2 |
| 23 | GO:0031643 | Positive regulation of myelination | 1.37E-03 | 9.52 | MAG, MYRF |
| 24 | GO:0030517 | Negative regulation of axon extension | 3.57E-03 | 5.88 | MAG, PLP1 |

**Table S2. Signaling enrichment of aberrantly expressed mRNAs in the ceRNA network by gene set enrichment analysis (GSEA)**

| **ID** | **Signaling pathway** | **Size** | **ES** | **NES** | **P Value** | **Rank at max** |
| --- | --- | --- | --- | --- | --- | --- |
| **MAG** |  |  |  |  |  |  |
| 1 | **Glutathione metabolism** | 44 | 0.55 | 1.59 | <0.01 | 4146 |
| 2 | Adherens junction | 72 | 0.38 | 1.58 | <0.01 | 1627 |
| 3 | Nicotinate and nicotinamide metabolism | 22 | 0.59 | 1.53 | <0.01 | 6014 |
| 4 | RIG I like receptor signaling pathway | 60 | 0.45 | 1.48 | <0.01 | 6707 |
| 5 | Prostate cancer | 85 | 0.40 | 1.42 | <0.01 | 6480 |
| 6 | Sphingolipid metabolism | 38 | 0.52 | 1.66 | 0.024 | 440 |
| 7 | Abc transporters | 44 | 0.51 | 1.28 | 0.035 | 7853 |
| 8 | Pathways in cancer | 312 | 0.36 | 1.35 | 0.036 | 5645 |
| 9 | Pyrimidine metabolism | 95 | 0.33 | 1.36 | 0.038 | 6219 |
| 10 | Melanoma | 65 | 0.37 | 1.30 | 0.049 | 6480 |
| 11 | Protein export | 23 | -0.60 | -1.66 | <0.01 | 6961 |
| 12 | N-glycan biosynthesis | 46 | -0.32 | -1.45 | 0.035 | 6284 |
| 13 | Long term depression | 66 | -0.40 | -1.43 | 0.041 | 3849 |
| **HOXB3** |  |  |  |  |  |  |
| 1 | Nitrogen metabolism | 22 | -0.53 | -1.57 | <0.01 | 1840 |
| 2 | O-glycan biosynthesis | 26 | -0.60 | -1.52 | <0.01 | 4072 |
| 3 | Glutathione metabolism | 44 | -0.51 | -1.50 | <0.01 | 1842 |
| 4 | Tyrosine metabolism | 38 | -0.48 | -1.42 | <0.01 | 1965 |
| 5 | Melanoma | 65 | -0.41 | -1.37 | 0.020 | 1978 |
| 6 | Tryptophan metabolism | 35 | -0.43 | -1.33 | 0.023 | 743 |
| 7 | Sphingolipid metabolism | 38 | -0.54 | -1.63 | 0.023 | 3766 |
| 8 | Alanine aspartate and glutamate metabolism | 30 | -0.48 | -1.52 | 0.025 | 1969 |
| 9 | Glyoxylate and dicarboxylate metabolism | 16 | -0.49 | -1.43 | 0.041 | 4774 |
| 10 | Long term depression | 66 | 0.46 | 1.77 | <0.01 | 8396 |
| 11 | Linoleic acid metabolism | 22 | 0.73 | 1.52 | 0.038 | 2273 |
| **MYRF** |  |  |  |  |  |  |
| 1 | Glutathione metabolism | 44 | 0.55 | 1.60 | <0.01 | 4146 |
| 2 | Adherens junction | 72 | 0.38 | 1.55 | <0.01 | 1627 |
| 3 | Nicotinate and nicotinamide metabolism | 22 | 0.59 | 1.53 | <0.01 | 6014 |
| 4 | RIG I like receptor signaling pathway | 60 | 0.45 | 1.43 | <0.01 | 6707 |
| 5 | Prostate cancer | 85 | 0.40 | 1.42 | <0.01 | 6480 |
| 6 | Abc transporters | 44 | 0.51 | 1.31 | 0.027 | 7853 |
| 7 | Pyrimidine metabolism | 95 | 0.33 | 1.35 | 0.029 | 6219 |
| 8 | Pathways in cancer | 312 | 0.36 | 1.31 | 0.037 | 5645 |
| 9 | Sphingolipid metabolism | 38 | 0.52 | 1.62 | 0.041 | 440 |
| 10 | Protein export | 23 | -0.60 | -1.61 | <0.01 | 6961 |
| 11 | N-glycan biosynthesis | 46 | -0.32 | -1.42 | 0.024 | 6284 |
| **PLP1** |  |  |  |  |  |  |
| 1 | Glutathione metabolism | 44 | 0.55 | 1.58 | <0.01 | 4146 |
| 2 | Adherens junction | 72 | 0.38 | 1.56 | <0.01 | 1627 |
| 3 | Nicotinate and nicotinamide metabolism | 22 | 0.59 | 1.52 | <0.01 | 6014 |
| 4 | RIG I like receptor signaling pathway | 60 | 0.45 | 1.47 | <0.01 | 6707 |
| 5 | Prostate cancer | 85 | 0.40 | 1.41 | <0.01 | 6480 |
| 6 | Pathways in cancer | 312 | 0.36 | 1.33 | 0.027 | 5645 |
| 7 | Sphingolipid metabolism | 38 | 0.52 | 1.65 | 0.033 | 440 |
| 8 | Pyrimidine metabolism | 95 | 0.33 | 1.36 | 0.033 | 6219 |
| 9 | Abc transporters | 44 | 0.51 | 1.29 | 0.040 | 7853 |
| 10 | Melanoma | 65 | 0.37 | 1.31 | 0.043 | 6480 |
| 11 | Protein export | 23 | -0.60 | -1.65 | <0.01 | 6961 |
| 12 | N-glycan biosynthesis | 46 | -0.32 | -1.42 | 0.031 | 6284 |

**ES**, Enrichment score; **NES**, normalized enrichment score.
